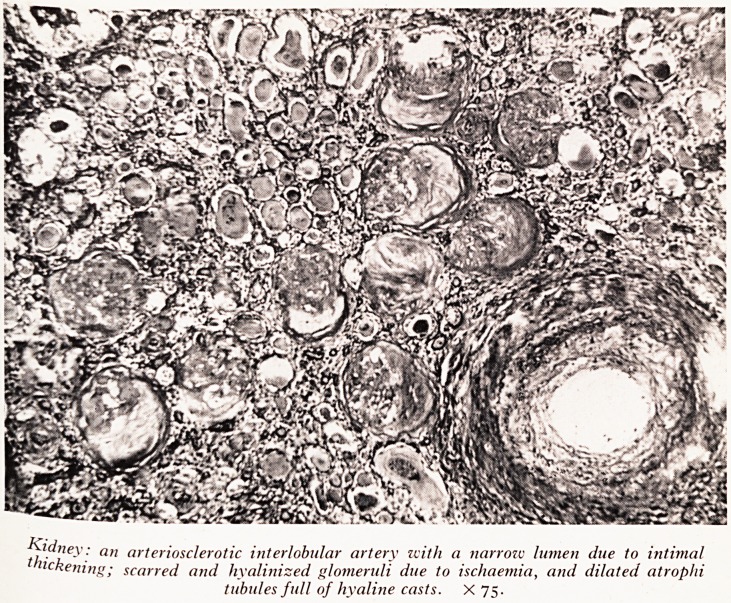# Renal Complications of Diabetes Mellitus

**Published:** 1960-03

**Authors:** T. F. Hewer


### "RENAL COMPLICATIONS OF DIABETES MELLITUS"

*A Clinical Pathological Conference of the University of Bristol Medical School*
(*P.M.* 6169)

CHAIRMAN: PROFESSOR T. F. HEWER

*Professor Hewer:* I will ask Dr. J. E. Cates to give us the clinical history of this patient.

*Dr. J. E. Cates:* Miss L. W. aged 55, a known diabetic, was admitted to Bristol Royal Infirmary on the 11th May, 1959, and died in uraemia three days later. Diabetes was diagnosed first in 1951 shortly after the death of her mother. We have no details of her treatment at that time, except that she was not given insulin. She first came to the Bristol Royal Infirmary as an out-patient in May 1955, referred by her family doctor with the diagnosis of diabetes, and with a report that her urine contained a trace of sugar, much albumin and some pus. The doctor also reported that whereas six years ago her blood pressure was 165/90 it had risen now to 205/105. Apart from the usual symptoms of diabetes, she complained of ankle swelling and shortness of breath. She was very fat and was therefore given a low calorie diet which caused her to lose 14 lb. in weight; her urine became sugar-free, and her blood sugar fell to normal levels. Her urinary infection was due to *Bacillus coli* which was sensitive to sulphonamides, tetracycyline, chloramphenicol and streptomycin; and granular casts were also noted in her urine. She was given a course of sulphonamides. The urinary infection was not eradicated and she defaulted from the clinic. She also had early cataracts, and was referred to Bristol Eye Hospital for opinion.

Three years later (September 1958), she was referred to the Diabetic Clinic from the Eye Hospital because her diabetes was not fully controlled. She was now complaining of increasing breathlessness, orthopnea, and some chest pain on exertion. She was still very fat, her blood pressure had now risen to 260/140 with an increase in her heart size and a palpable gallop rhythm. There was ankle oedema and râles at her bases. There was also diabetic neuropathy with absent ankle jerks and reduced sensitivity to pin prick below the knee. In her fundi there were excessive haemorrhages and exudates, and a blurring of her disc margins. The urine still contained much albumin, pus and a heavy growth of *Bacillus coli.* Her blood urea was now raised (100 mg. per cent). There was very little glycosuria; and her blood sugar was 160 mg. per cent two hours after food. Her practitioner was again asked to prescribe a course of sulphonamides but advised not to give potassium citrate. She attended at fortnightly intervals and was prescribed a low calorie diet on which she lost weight, and her blood urea rose to 122 mg. per cent. She was advised to come into hospital for more vigorous treatment of her urinary infection but declined and once more defaulted.

In May 1959 she was admitted as an emergency with a three-day history of passing no urine at all. Two weeks before this she had felt unwell, nauseated and had vomited once or twice. Her skin had begun to itch and for the first time she had burning and smarting on micturition, with a little blood in her urine. At the same time she had a sore throat, and reported that her doctor had given her tablets.

On examination she was now grossly oedematous, and was overbreathing. Her breath smelt uraemic and foul. Her mouth contained stale blood from her bleeding gums. The other physical findings were as before, except that her blood pressure had fallen to 145/70; blood sugar 160 mg. per cent. Throat swab showed no growth of haemolytic streptococci. Blood N.P.N. 246 mg. per cent; sodium 128 m. eq.; potassium 6·3 m. eq. Catheterization of her bladder yielded no urine. The next day Mr. Ashton Miller catheterized her right ureter and found no obstruction up to 25 cm.; the left ureteric orifice was not seen. On the 13th May N.P.N. 326, K. 7·4 m. eq. alkali reserve 8 m. eq. She was treated with intravenous tetracycline and penicillin and was given resonium by stomach tube together with glucose. She died in coma the next day.

We felt that this lady had chronic pyelonephritis, probably with an acute flare-up of recent onset, over the previous two weeks; the heavy albuminuria throughout suggested that her kidneys were probably affected by the diabetic glomerulo-sclerosis described by Kimmelstiel and Wilson.

There were two other probabilities that needed excluding: one was that she might have developed papillitis necroticans and that a papilla might have sloughed off and blocked her ureter. Mr. Ashton Miller helped to exclude this by his ureteric catheterization. The second possibility was that her family doctor had given her sulphonamide again and that she had developed anuria from this, but we learned soon after admission that he had prescribed hydrochlorthiazide and Rauwiloid.

*Question:* Was an artificial kidney available and would this have been a valuable treatment ?

*Dr. Cates:* No, it was not available. Even if it had been, I should not have used it because all the evidence indicated that this woman's kidneys were permanently and severely damaged. An artificial kidney is of value for tiding people over a period of temporary renal insufficiency.

*Question:* What are your criteria for deciding that a kidney lesion is irreversible ?

*Dr. Cates:* From the history of her slowly developing impairment of renal function, and from our failure to find a reversible condition.

*Question:* How was it that the blood pressure fell ? Could this have aggravated her renal failure ?

*Dr. Cates:* It is certainly true that a fall in blood pressure might have decreased glomerular filtration. We did not discover a separate cause for this fall in blood pressure; blood pressure often falls in the closing stages of uraemia.

*Question:* Would Dr. Cates like to comment on the value of sulphonamide in the treatment of pyelonephritis ?

*Dr. Cates:* The weight of evidence is that sulphonamides are not likely to eradicate the infection within the kidney substance in pyelonepritis; for some years now I have preferred to give large doses of antibiotics for ten days or more at a time, almost on the scale of that needed in B. coli endocarditis.

*Question:* Would Dr. Cates condemn the use of sulphonamides in all cases of urinary infection ?

*Dr. Cates:* Oh no. In an acute cystitis, after catheterization for example, sulphonamides may be enough. But even then you ought to check the urine after a few weeks to see if it is still clear.

*Dr. H. M. Leather:* If the blood urea is high it is a safe rule not to use sulphonamides.

*Dr. Cates:* I agree.

*Dr. W. O. Spence:* Would Dr. Cates mind repeating what he said about the use of citrate in renal insufficiency ?

*Dr. Cates:* It is the potassium that is dangerous if renal function is impaired. The citrate radicle is innocuous.

*Question:* Did diabetic coma play any part in this woman's illness ?

*Dr. Cates:* No; though the obvious acidosis raised the possibility. But her blood sugar was normal and we couldn't detect ketones in her breath. In doubtful cases where there is no urine you can use a Rothera test on plasma.

*Professor Hewer:* I think I probably ought to give the post mortem findings at this stage. This was a very obese woman who died with some oedema of the lungs and a bilateral pleural effusion. The heart, which weighed 585 grams, had considerable left ventricular hypertrophy and some hypertrophy of the right ventricle as a result, no doubt, of recurrent attacks of left ventricular insufficiency. There happened to be a

PLATE III

*Kidneys.*

PLATE IV

*A segment of ureter showing several mucous cysts projecting into lumen.* × 3.

PLATE V

*Photomicrograph of section of ureter showing a small cyst and, on the left, a mucous gland becoming dilated.* × 70.

PLATE VI

*Kidney: glomeruli with some ischaemic shrinkage and large hyaline Kimmelstiel-Wilson lesions.* × 75.

PLATE VII

*Kidney: an arteriosclerotic interlobular artery with a narrow lumen due to intimal thickening; scarred and hyalinized glomeruli due to ischaemia, and dilated atrophi tubules full of hyaline casts.* ×75.

congenitally bicuspid aortic valve but this had not contributed to her cardiac embarrassment and there was no other change in the heart. There was a good deal of atheroma in the aorta but the renal arteries in particular were very atheromatous and the branches to the right kidney were extremely narrow. As you will have gathered the kidneys were the most interesting part of the necropsy. The left kidney was much larger than the right weighing 220 grams while the right weighed only 115 (Plate III). This difference was due largely to a considerable shrinkage of the upper half of the right kidney as a result of extreme atheromatous narrowing of the branches of the renal artery supplying that area. In all other respects the two kidneys were identical, the capsule stripping with some difficulty leaving a granular surface. The general pattern of the left kidney on section was reasonably normal with diffuse scarring and just a little dilatation of the pelvis. The pelvis of each kidney showed a few small cysts projecting on the surface and these cysts were present throughout the length of both ureters (Plate IV), projecting like a series of minute balloons full of mucus. They must have caused quite appreciable obstruction to the flow of urine down the ureters. The condition is that of pyelitis cystica and ureteritis cystica. As will be seen in the photograph (Plate V) these cysts are due to dilatation, as a result of obstruction, of small mucous glands beneath the epithelium lining the ureters. This state of affairs is sometimes found in chronic inflammation of the urinary tract. It is occasionally present in the urinary bladder also but in this case there was only a mild subacute cystitis with no cystic change.

The microscopic appearance of these kidneys was quite remarkable. Several different conditions were present, all of them being quite frequent accompaniments of chronic diabetes. Firstly there was the condition of glomerulosclerosis of Kimmelstiel and Wilson. Nearly every glomerulus, in both kidneys, had in the substance of the glomerular tuft one or more round hyaline bodies which stained pink with eosin and gave a fine fibrillar staining with silver impregnation for reticulin (Plate VI). These lesions develop in the wall of the capillary vessels of the glomerulus. They contain little or no lipid. They undoubtedly give rise to a considerable reduction in blood flow through the glomerulus. Another change in all of the glomeruli was the presence of a quite uniform fibrous thickening of the walls of all the capillary loops in the glomeruli amounting to a diffuse glomerulosclerosis. A third manifestation of glomerular damage, which, like the Kimmelstiel-Wilson lesion, is pathognomonic of diabetes, was an exudative lesion at the periphery of the glomerulus consisting of a very acidophil vesicular lesion which, in frozen sections, contained anisotropic lipid. This is the exudative lesion which was described by Dr. G. F. M. Hall (1952) of this Department.

In addition to these glomerular lesions all of the afferent arterioles were very much thickened and completely hyalinized as a result of the hypertension. All of the branches of the renal artery within the kidney showed a considerable degree of narrowing as a result of intimal proliferation and atheroma. In the right kidney the area which was supplied by the very much narrowed renal arteries had shrunken as a result of ischaemia and it was interesting to see that the glomeruli in the ischaemic area were almost totally scarred but still showed signs of having had the Kimmelstiel-Wilson nodular lesions. This is interesting in that it is evidence that the Kimmelstiel-Wilson lesion appeared before the renal artery narrowing gave rise to such severe ischaemia.

In both kidneys there were some tubules containing polymorphonuclear leucocytes which can be interpreted as evidence of a slight pyelonephritis in an active stage. In the more scarred parts of both kidneys were numerous greatly atrophied renal tubules which were dilated and full of protein material giving them the appearance of thyroid tissue. This is rather characteristic of chronic pyelonephritis (Plate VII).

Another lesion of some interest in the kidney was the presence of some necrosis of the epithelium of the collecting tubules in the medulla together with a number of desquamated cells lying in the lumen. This necrosis of tubule epithelium is called lower nephron nephrosis and it is found in a variety of conditions where the renal blood flow is drastically reduced. I think you will agree that there are plenty of reasons in this case for such a reduction of blood flow and when one remembers that the blood pressure of this patient fell considerably towards the end it is not surprising that lower nephron nephrosis occurred. By this time undoubtedly renal blood flow was so reduced that there was practically no glomerular filtration and this explains the anuria.

Mention has been made by Dr. Cates of the possibility of renal papillary necrosis as a contributory factor in causing the anuria in this case. Some of the calyces in the ischaemic part of the right kidney were dilated and it looked at first sight as though the tips of the pyramids had in fact disappeared, but careful histological examination showed no evidence of any old papillitis necroticans.

We can conclude, therefore, that these kidneys suffered from several conditions, all of which contributed to a reduction in the renal blood flow and glomerular filtration. There was a generalized glomerulosclerosis, with the nodular Kimmelstiel-Wilson lesions, an exudative type of glomerulosclerosis of diabetic origin, arteriolar sclerosis resulting from the hypertension, general renal artery narrowing produced by atherosclerosis, and some obstruction to the ureters by the condition of ureteritis cystica. There was also a mild pyelonephritis. Lastly one might mention tubular necrosis which may well have been due to the fall of blood pressure.

Changes in other organs were not of great significance. There was a generalized arteriolar hyalinization as a result of hypertension. The uterus contained numerous fibroids and some endometrial polyps. There were no obvious lesions in the islets of Langerhans.

It is quite clear that all the renal lesions that I have described were amply sufficient to account for the renal failure and I am sure that Dr. Cates was fully justified in considering that this woman's renal state was irrecoverable.

*Question:* Did you give the patient any insulin ?

*Dr. Cates:* No; she was a fat mild diabetic who readily responded to dietary restriction: she lost her glycosuria, and her blood sugars were virtually normal when on this regime. I am not certain, however, whether she ought not to have had a little insulin in view of her retinopathy. What does Dr. Mather think about that ?

*Dr. H. G. Mather:* I don't think insulin does make any difference in a case like this.

*Professor Hewer:* There is little more to be said about this patient. It is clear that she exemplified most of the renal lesions that occur in chronic diabetes.

### REFERENCE

Hall, G. F. M. "The significance of atheroma of the renal arteries in Kimmelstiel-Wilson's Syndrome" *J. Path. & Bact.*, **64**, 103–120.

## Figures and Tables

**Figure f1:**
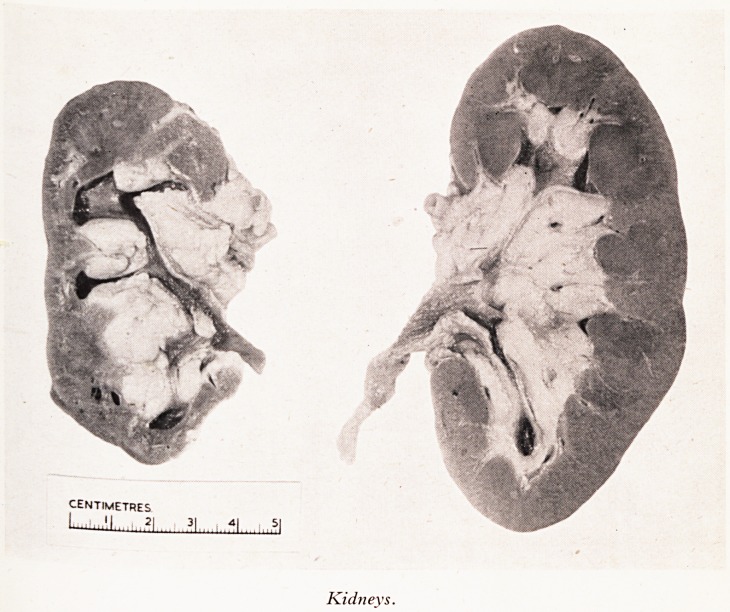


**Figure f2:**
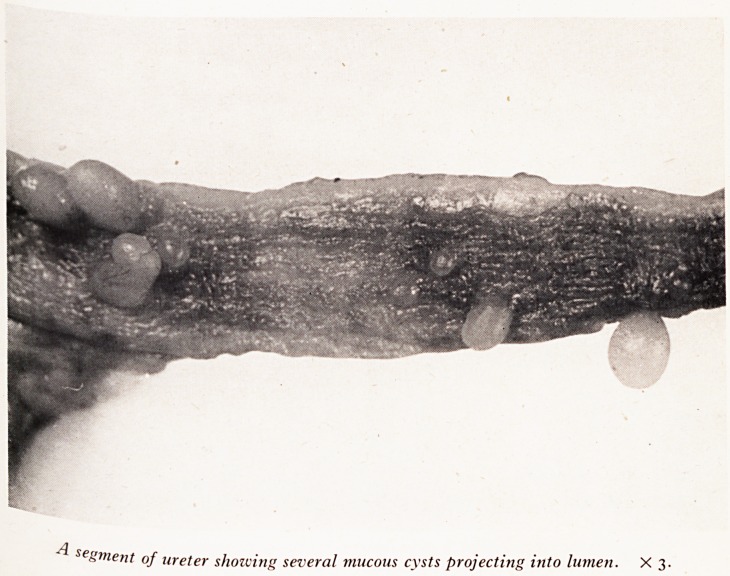


**Figure f3:**
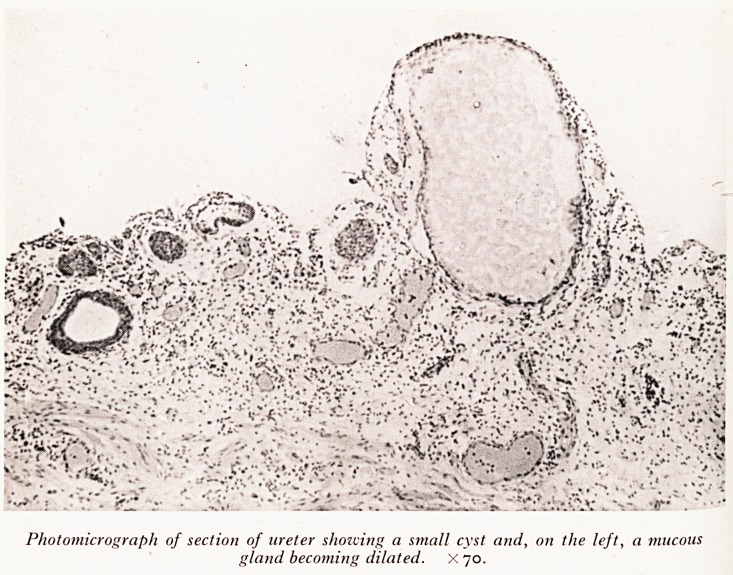


**Figure f4:**
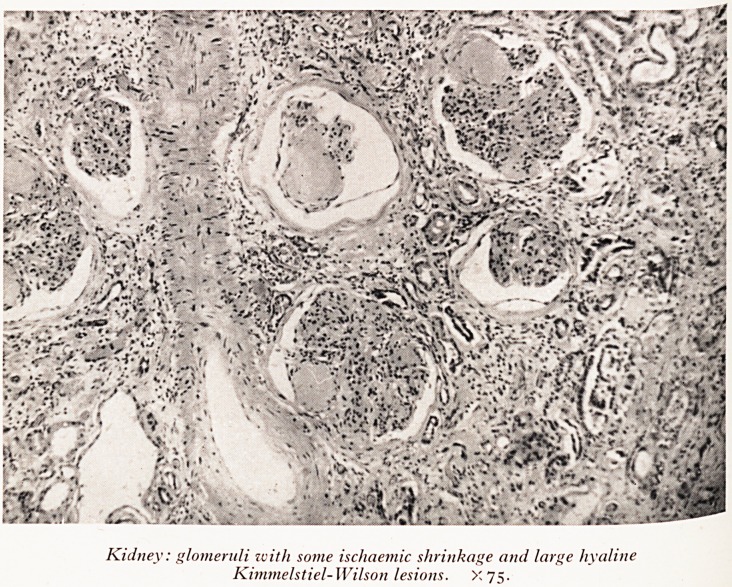


**Figure f5:**